# Downregulation of lncRNA ANRIL Inhibits Osteogenic Differentiation of Periodontal Ligament Cells via Sponging miR-7 through NF-*κ*B Pathway

**DOI:** 10.1155/2021/7890674

**Published:** 2021-11-24

**Authors:** Xinwei Liu, Yue Zhou

**Affiliations:** Department of Stomatology, Beihua University Affiliated Hospital, Jilin 132021, China

## Abstract

**Background:**

Long noncoding RNAs (lncRNAs) are dysregulated in periodontitis development and involved in osteogenesis. The current study was aimed at investigating the function of lncRNA ANRIL in periodontal ligament cells (PDLCs) and potential molecular mechanisms.

**Methods:**

Firstly, the level of ANRIL was tested by qPCR. Then, PDLCs were treated with a mineralizing solution to induce osteogenic differentiation. ALP activity was measured, and protein levels of BMP2, Osterix, and OCN were measured by Western blot. A target of ANRIL was verified using dual-luciferase reporter assay. miR-7 level was measured by qPCR, and the signals of the NF-*κ*B pathway were tested by Western blot.

**Results:**

ANRIL expression was downregulated in PDL tissues. Next, ALP activity and protein levels of BMP2, Osterix, and OCN were increased to show that PDLCs were differentiated. ANRIL level was increased in differential PDLCs, in which knockdown inhibited osteogenic differentiation. Then, miR-7 was found as a target of ANRIL. The miR-7 level was upregulated in PDL tissues and reduced in differential PDLCs. Inhibition of miR-7 suppressed ALP activity and BMP2, Osterix, and OCN expression. Moreover, inhibition of miR-7 reversed the effects on the osteogenic differentiation induced by knockdown of ANRIL. Besides, the levels of p-P65 and p-I*κ*B*α* were elevated by ANRIL downregulation and were rescued by suppressing miR-7.

**Conclusions:**

Knockdown of ANRIL inhibited osteogenic differentiation via sponging miR-7 through the NF-*κ*B pathway, suggesting that ANRIL might be a therapeutic target for periodontitis.

## 1. Background

Periodontitis is a chronic, nonspecific, and multifactorial inflammatory disease associated with periodontal support tissue. It will cause pathological loss of the periodontal ligament and alveolar bone, leading to teeth loss ultimately [1]. Diabetes, obesity, and aging are associated with the pathogenic factors of periodontitis [2–4]. Periodontitis may be a potential risk factor for other human diseases like Alzheimer's disease and stroke, which affect human systemic health [5, 6]. The periodontal ligament (PDL), made up of collagen fiber bundles and cells, has many functions, including tooth support, tooth nutrition, alveolar bone remodeling, and damage tissue repair [7]. Periodontal ligament cells (PDLCs) play essential roles in maintaining the homeostasis of periodontal tissue and repairing periodontal ligament. Unfortunately, periodontitis injures the osteogenic differentiation of PDLCs [8]. The treatment of periodontitis is complex, and there is still a lack of early screening biomarkers and therapeutic targets.

Long noncoding RNAs (lncRNAs) are a class of more than 200 nt noncoding transcripts. Recently, with the gradual deepening of lncRNA's biological and functional roles, we found that lncRNA acts as miRNA sponges and then regulates mRNA effects [9]. An increasing body of evidence has suggested that lncRNAs are involved in human diseases, such as cardiovascular diseases [10], malignant tumors [11], and inflammation-related diseases [12]. As important inflammatory regulators, lncRNAs are often abnormally expressed in the progression of periodontitis [13] and associated with osteogenesis [14]. An antisense lncRNA, antisense noncoding RNA in the INK4 locus (ANRIL), is located at the CDKN2A/B genomic locus, consisting of at least 21 exons and a large number of reported isoforms [15]. ANRIL is reported as an immune response-related lncRNA, in which expression is reduced in the peripheral blood of patients with periodontitis [16]. However, biological functions are still largely unknown.

In the present study, the effects of ANRIL on the osteogenic differentiation of PDCLs were explored. Moreover, ANRIL was found to be sponging to miR-7. Notably, the mechanism of ANRIL sponged to miR-7 to regulate the osteogenic differentiation of PDLCs was investigated.

## 2. Methods

### 2.1. PDL Tissue Collection

A total of 30 patients with periodontitis and 30 healthy controls participated in the study. This study protocol was approved by the Ethics Committee of Beihua University Affiliated Hospital.

Written informed consent was provided before the study. All participators were diagnosed with periodontitis or not by Beihua University Affiliated Hospital. None of them had infectious diseases, a history of smoking, and orthognathic surgery. At routine premolar or third molar extractions, the PDL tissues were separated from the middle 1/3 of the dental roots. Partial tissues were stored at -80°C until further use.

### 2.2. Cell Culture and Osteogenic Induction

Other tissues were cut into 1 mm^3^ pieces and digested by 3 mg/ml of collagenase type I (Sigma-Aldrich, USA) and 4 mg/ml of dispase (Corning, USA) at 37°C. The cell suspension was maintained in DMEM (Hyclone, USA) supplemented with 10% FBS (Solarbio, China) and 1% penicillin/streptomycin (Solarbio, China) at 37°C with 5% CO_2_. The medium was changed every 2-3 days until cell passage to the fifth generation. For osteogenic induction, PDLCs were seeded into 6-well plates until the confluency researched exceed 70%. DMEM supplemented with 10% FBS, 10 mM *β*-glycerophosphate (Sigma-Aldrich, USA), 50 *μ*g/ml vitamin C (Aladdin, China), and 10 nM dexamethasone (Aladdin, China) was used as an osteogenic-induced medium. Two weeks postincubation, PDLCs were harvested for testing osteogenic differentiation.

### 2.3. Alkaline Phosphatase (ALP) Activity Analysis

ALP activity was assessed by the ALP Assay Kit (Beyotime, China). Briefly, PDLCs were lysed by a lysis buffer and seeded into 96-well plates. The test buffer was added and incubated with cells at 37°C for 10 min. After stopping the reaction, the absorbance was measured at 405 nm.

### 2.4. Dual-Luciferase Reporter Assay

The sequences of ANRIL containing the miR-7 potential binding sites were amplified and inserted into pGL3 vectors (Promega, USA) as the ANRIL-WT group. The ANRIL-MUT group was obtained by targeted mutation. miR-7 mimic and mimic negative control (NC) were purchased from GenePharma (Shanghai, China). HEK293T cells were seeded into 24-well plates and cotransfected with ANRIL-WT or ANRIL-MUT and miR-7 mimic or mimic NC using Lipofectamine 2000 (Invitrogen, USA). After 24 h, the relative luciferase activity (firefly activity normalized to Renilla activity) was measured by Dual-Luciferase Reporter Assay Kit (Promega, USA).

### 2.5. Cell Transfection

shRNA-NC, shRNA-ANRIL, miR-7 inhibitor, and inhibitor-NC were all acquired from GenePharma (Shanghai, China). PDLCs in the logarithmic growth phase were seeded into 6-well plates, and the transfection process used Lipofectamine 2000 (Invitrogen, USA). After 48 h, transfection efficiency was detected.

### 2.6. qPCR

Total RNA was isolated from PDLCs by TRIzol reagent (Sigma-Aldrich, USA). After concentration and purity testing, RNA was reverse transcribed into cDNA using LnRcute lncRNA cDNA First Chain Synthetic Kit (Tiangen, China), and miRNA reverse transcription was conducted by miScript II RT Kit (Qiagen, Germany). INRcute lncRNA qCPR Detection Kit (SYBR Green) (Tiangen, China) was performed for qPCR of lncRNA with the following conditions: 95°C for 3 min, 40 cycles of 95°C for 5 sec, and 60°C for 15 sec. qPCR was used to measure miR-7 level by microRNA qPCR kit (SYBR Green Method) (Sango, China) with the conditions as 95°C for 30 sec, 95°C for 5 sec, and 60°C for 30 sec (40 cycles). The level of mRNA was detected by Real-Time One-Step RT-qPCR (Tiangen, China) for reverse transcription and qPCR, and the conditions were 50°C for 30 min, 95°C for 3 min, 40 cycles of 95°C for 15 sec, and 60°C for 30 sec. The reaction instrument was an ABI PRISM 7500 system (Applied Biosystems, USA). *β*-Actin level was performed as the loading control. The results of relative expression were assessed by the 2 − ΔΔCt.

### 2.7. Western Blot

The transfected cells were collected, and precooled RIPA lysate (Beyotime, China) was added to extract the total protein. After 10% SDS-PAGE, the protein was transferred to PVDF membranes (Millipore, USA) and blocked with 5% skim milk. Primary antibodies including anti-BMP2, anti-Osterix, anti-osteocalcin (OCN), anti-P65, anti-p-P65, anti-I*κ*B*α*, and anti-p-I*κ*B*α* were added and incubated with the membranes at 4°C overnight. After washing the membranes, the secondary antibody was added to incubate at room temperature for 1 h. The protein bands were developed by ECL Western Blotting Substrate (Pierce, USA) and then photographed. The gray analysis was performed by ImageJ software 1.48U (Bethesda, USA).

### 2.8. Statistical Analysis

The results in this study were analyzed by GraphPad Prism 6.0 (GraphPad Software, USA) and presented as mean ± standard deviation (SD). Student's *t*-test was used for multiple comparisons between two groups, and one-way ANOVA was used between three or more groups. *P* < 0.05 was deemed to have significant differences.

## 3. Results

### 3.1. The Level of ANRIL Was Downregulated in Periodontitis

**Figure 1:**
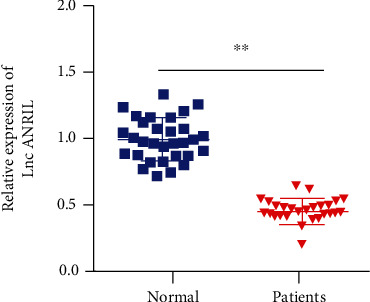
The expression of ANRIL was decreased in PDL tissues of periodontitis patients. PDL tissues from 30 patients with periodontitis and 30 healthy people were collected, and then, ANRIL level was detected by qPCR. ^∗∗^*P* < 0.01.

Firstly, the PDL tissues were obtained from periodontitis patients and healthy people, and the expression of ANRIL was measured. According to the results of qPCR, the ANRIL level was reduced in PDL tissues of patients with periodontitis, compared with the healthy control group (Figure 1).

### 3.2. Identification of the Osteogenic Differentiation of PDLCs

**Figure 2:**
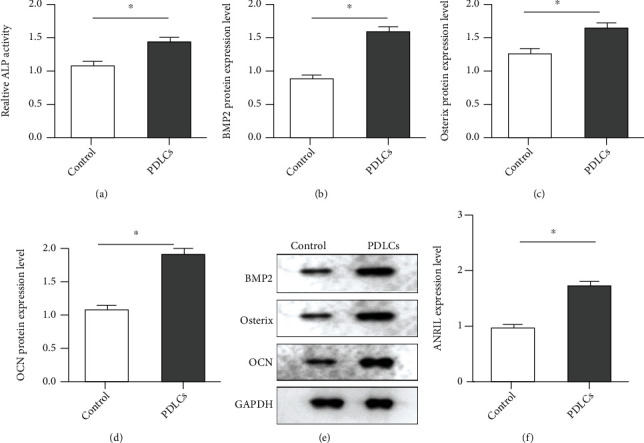
Identification of osteogenic differentiation model. (a) ALP activity was detected in undifferentiated and differentiated PDLCs. The relative protein expression normalized to GAPDH was calculated, including (b) BMP2, (c) Osterix, and (d) OCN. (e) The protein levels of osteogenic-related markers as BMP2, Osterix, and OCN were tested by Western blot. GAPDH was performed as the housekeeping control. (f) The expression of ANRIL was measured in undifferentiated and differentiated PDLCs by qPCR. ^∗^*P* < 0.05.

To investigate the osteogenic differentiation capability, PDLCs derived from PDL tissues were cultured in DMEM with *β*-glycerophosphate, vitamin C, and dexamethasone. Subsequently, some of the specific markers associated with osteogenesis were evaluated. As illustrated in Figure 2(a), ALP activity was elevated in osteogenic differentiated PDLCs. The protein levels of BMP2, Osterix, and OCN were all upregulated in PDLCs after treatment (Figures 2(b)–2(e)). Moreover, the expression of ANRIL was upregulated in differentiated PDLCs, compared with undifferentiated PDLCs (Figure 2(f)).

### 3.3. Knockdown of ANRIL Inhibited Osteogenic Differentiation of PDLCs

**Figure 3:**
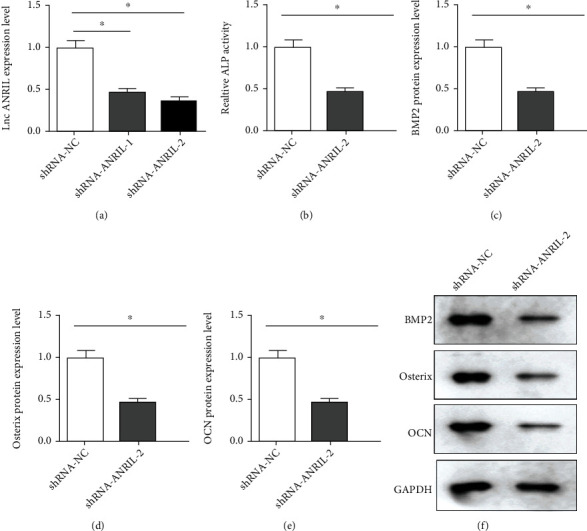
(a) The transfection efficiency was measured by qPCR with PDLC transfection of shRNA-ANRIL-1, shRNA-ANRIL-2, and shRNA-NC. (b) ALP activity was detected posttransfection. The protein levels of (c) BMP2, (d) Osterix, and (e) OCN were quantified. (f) The proteins were detected by Western blot posttransfection. GAPDH was used as an internal control. ^∗^*P* < 0.05.

To explore the biological functions of ANRIL in PDLCs, inhibition of ANRIL expression was conducted by transfection of shRNA-ANRIL-1 and shRNA-ANRIL-2. To examine the transfection efficiency, qPCR was conducted. Compared with the shRNA-NC group, the level of ANRIL was downregulated in the shRNA-ANRIL-1 and shRNA-ANRIL-2 groups, especially in the shRNA-ANRIL-2 group (Figure 3(a)). To evaluate the effects of ANRIL on osteogenic differentiation, ALP activity was measured. As shown in Figure 3(b), shRNA-ANRIL-2 suppressed the ALP activity, compared with shRNA-NC (Figure 3(b)). Additionally, the protein expression of BMP2, Osterix, and OCN was tested by Western blot, and the results demonstrated that knockdown of ANRIL repressed BMP2, Osterix, and OCN levels (Figures 3(c)–3(f)).

### 3.4. miR-7 Was Identified as a Target of ANRIL

**Figure 4:**
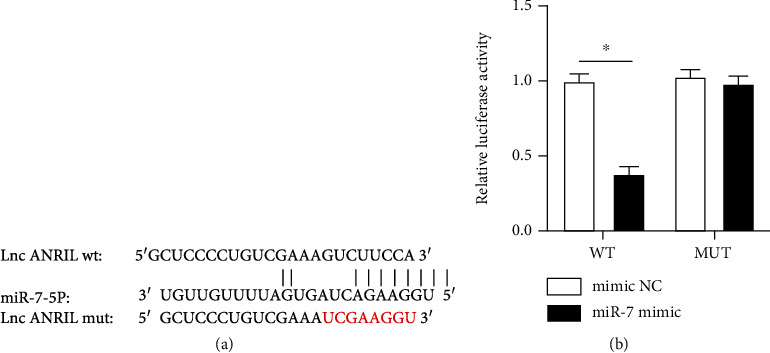
ANRIL sponged to miR-7. (a) The binding sites between ANRIL and miR-7 are shown. The mutant sequences of ANRIL are also shown. (b) HEK293T cells were cotransfected with ANRIL-WT or ANRIL-MUT together with miR-7 mimic or mimic NC, and the relative luciferase activity was detected. ^∗^*P* < 0.05.

The binding sites between ANRIL and miR-7 are shown in Figure 4(a). To verify the targeted relationship, a dual-luciferase reporter assay was conducted. The results demonstrated that the relative luciferase activity was decreased in HEK293T cells cotransfected with ANRIL-WT and miR-7 mimic, compared with cotransfection of ANRIL-WT and mimic NC. However, in the ANRIL-MUT group, there was no difference between the miR-7 mimic and the mimic NC (Figure 4(b)).

### 3.5. The Expression of miR-7 Was Evaluated in PDL Tissues and PDLCs

**Figure 5:**
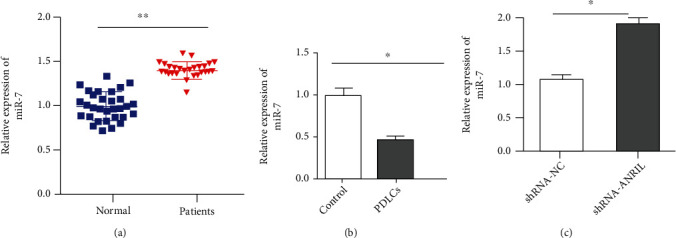
miR-7 expression in PDL tissues and PDLCs. (a) The level of miR-7 was tested in PDL tissues from periodontitis patients and healthy control by qPCR. (b) miR-7 level was detected by qPCR in undifferentiated and differentiated PDLCs. (c) The expression of miR-7 was measured by qPCR after PDLCs were transfected with shRNA-NC and shRNA-ANRIL-2. ^∗^*P* < 0.05; ^∗∗^*P* < 0.01.

Subsequently, the expression of miR-7 was measured. As compared to healthy control tissues, the miR-7 level was upregulated in PDL tissues (Figure 5(a)). Additionally, the miR-7 level was decreased in differentiated PDLCs, compared with undifferentiated PDLCs (Figure 5(b)). After knockdown of ANRIL, the expression of miR-7 was elevated, compared with the shRNA-NC group (Figure 5(c)).

### 3.6. Downregulation of miR-7 Promoted Osteogenic Differentiation of PDLCs

**Figure 6:**
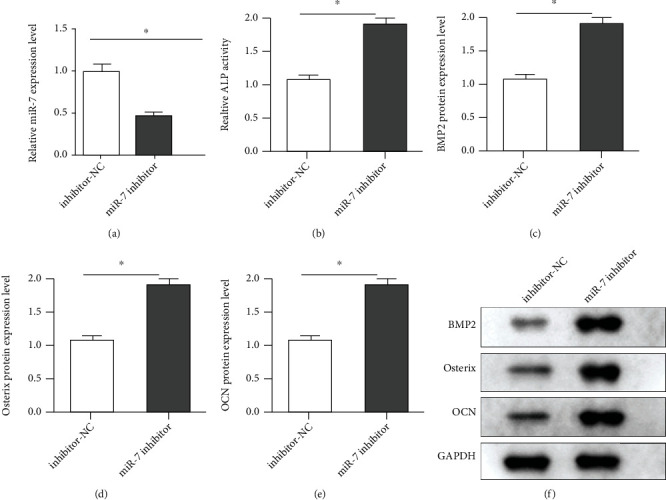
miR-7 downregulation facilitated PDLC osteogenic differentiation. (a) The transfection efficiency was measured by qPCR in PDLC transfection of miR-7 inhibitor and inhibitor-NC. (b) After transfection, ALP activity was evaluated. The protein levels of (c) BMP2, (d) Osterix, and (e) OCN were quantified. (f) The proteins were assessed by Western blot. ^∗^*P* < 0.05; ^∗∗^*P* < 0.01.

For downregulation of miR-7, miR-7 inhibitor and inhibitor-NC were transfected into differentiated PDLCs. The data of transfection efficiency illustrated that the miR-7 level was reduced in the miR-7 inhibitor group, compared with the inhibitor-NC group (Figure 6(a)). Then, inhibition of miR-7 enhanced ALP activity and BMP2, Osterix, and OCN levels, compared with inhibitor-NC (Figures 6(b)–6(f)).

### 3.7. Knockdown of ANRIL Inhibited Osteogenic Differentiation through Sponging miR-7

**Figure 7:**
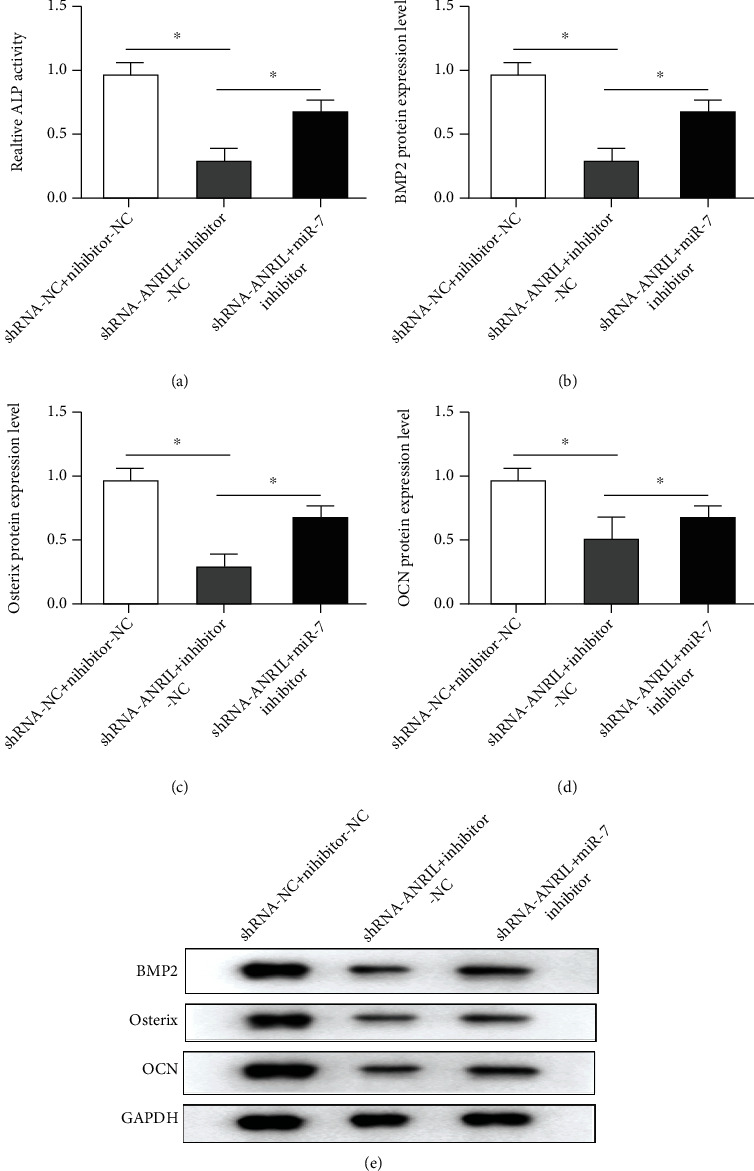
ANRIL knockdown suppresses osteogenic differentiation of PDLCs via regulating miR-7. (a) ALP activity was tested in PDLCs after transfection of shRNA-ANRIL-2 and miR-7 inhibitor. Gray analysis for Western blot of (b) BMP2, (c) Osterix, and (d) OCN protein levels. (e) The protein levels were assessed by Western blot. ^∗^*P* < 0.05.

ALP activity declined in the shRNA-ANRIL-2 group, which was not affected by inhibitor-NC but further abolished by miR-7 inhibitor (Figure 7(a)). Through the results of Western blot, knockdown of ANRIL inhibited BMP2, Osterix, and OCN levels. However, their levels were reversed by miR-7 downregulation (Figures 7(b) and 7(c)).

### 3.8. Knockdown of ANRIL Regulated NF-*κ*B Pathway by Mediating miR-7

**Figure 8:**
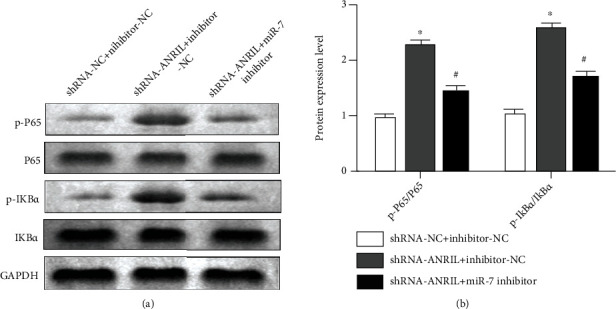
ANRIL activated NF-*κ*B pathway by inhibiting miR-7. (a) The relative protein expression of p-P65, P65, p-I*κ*B*α*, and I*κ*B*α* was quantified by normalizing to GAPDH level. (b) The protein of p-P65, P65, p-I*κ*B*α*, and I*κ*B*α* was examined by Western blot. ^∗^*P* < 0.05.

The protein expression of p-P65, P65, p-I*κ*B*α*, and I*κ*B*α* was measured by Western blot. The data demonstrated that knockdown of ANRIL enhanced the levels of p-P65 and p-I*κ*B*α*, which were rescued by inhibition of miR-7. However, both ANRIL and miR-7 did not affect P65 and I*κ*B*α* levels (Figures 8(a) and 8(b)).

## 4. Discussion

In the present study, we aimed to explore the biological functions of lncRNA ANRIL in osteogenic differentiation in PDLCs. We found that the level of ANRIL was downregulated in PDL tissues and upregulated in differential PDLCs. Knockdown of ANRIL inhibited ALP activity and BMP2, Osterix, and OCN levels, suggesting that downregulation of ANRIL inhibited osteogenic differentiation of PDLCs.

Recently, lots of researches revealed the role of ANRIL. Polymorphisms at the ANRIL gene are associated with the risks of many human diseases, including malignancy, cardiovascular disease, bone mass, obesity, and type 2 diabetes [15]. Generally, ANRIL is a prognostic biomarker and an oncomiR in human cancers, such as lung cancer, gastric cancer, and esophageal squamous cell carcinoma [17]. In addition, dysregulation of ANRIL promotes the development of atherosclerosis and leads to coronary heart disease through mediating single nucleotide polymorphisms and injuring the endothelial cell [18, 19]. Furthermore, ANRIL mediates VEGF which has an effect on diabetic retinopathy [20]. Osteogenesis is complex and plays a crucial role in periodontitis. Several lncRNAs have been reported to be involved in osteogenic differentiation, such as PCAT1 [21], MEG3 [22], TWIST1 [23], and MSC-AS1 [24]. lncRNA HOTAIR, controlled by the dental material methacrylate, can induce the HOXC11 gene to regulate osteoblast expression [25, 26]. Depletion of lncRNA MEG3 inhibits osteogenesis of PDLCs in periodontitis [27]. However, the roles of ANRIL in osteogenic differentiation, especially in PDLCs, are still unknown. The results of this study indicated that ANRIL downregulation had inhibited effects on the osteogenic differentiation of PDLCs.

Besides, the molecular mechanism of the differentiation induced by ANRIL was further investigated. miR-7 was regarded as a sponge of ANRIL. Zhao et al. have suggested that miR-7 abolishes the attenuation of oxidative injury of human trabecular meshwork cells induced by ANRIL [28]. Shu et al. have reported that silence of ANRIL exacerbates H9c2 cell injury induced by hypoxia by miR-7-5p/SIRT1 axis [29]. Li et al. have revealed that ANRIL mediates the migration and invasion of T-cell acute lymphoblastic leukemia via miR-7-5p/TCF4 axis [30]. In our study, we also verified the ANRIL sponge to miR-7 through the dual-luciferase reporter assay.

Accumulating evidence shows that miR-7 is involved in human diseases. For example, miR-7 may function as a tumor suppressor and regulate cellular processes, including growth, metastasis, differentiation, and apoptosis [31]. Besides cancers, abnormal expression of miR-7 is a potential biomarker for type 2 diabetes, acute pancreatitis, and Alzheimer's disease [32–34]. Moreover, miR-7 plays functional roles in osteoarthritis through regulating proliferation, apoptosis, and inflammation [35]. In periodontitis, miR-7 level was reduced during osteogenic differentiation, mediated by circular RNA ADR1as to repress osteoblastic differentiation of PDLCs [36]. In the present study, the results demonstrated that the expression of miR-7 was increased in PDL tissues and reduced in differential PDLCs. Downregulation of miR-7 promoted osteogenic differentiation of PDLCs. Moreover, knockdown of ANRIL inhibited osteogenic differentiation via sponging miR-7.

The noncanonical NF-*κ*B pathway is related to immune deficiencies, and abnormal activation of the pathway leads to the pathogenesis of a variety of autoimmune and inflammatory diseases [37]. Moreover, the NF-*κ*B pathway is also involved in osteogenic differentiation [38, 39]. Several studies have revealed that ANRIL and miR-7 mediate NF-*κ*B signaling. Overexpression of ANRIL facilitates angiogenesis of diabetes mellitus via activation of the NF-*κ*B pathway [40]. miR-7 suppresses pancreatic cancer progression through inactivation of the NF-*κ*B pathway [41]. In the present study, we found that knockdown of ANRIL enhanced the levels of p-P65 and p-I*κ*B*α*, which were rescued by inhibition of miR-7. These results suggested that downregulation of ANRIL activation inhibited osteogenic differentiation of PDLCs through the NF-*κ*B pathway by sponging miR-7.

## 5. Conclusions

The expression of ANRIL was decreased in PDL tissues, while the miR-7 level was increased. miR-7 was identified as a sponge of ANRIL. The level of ANRIL was elevated, and miR-7 was reduced in differentiated PDLCs. Importantly, knockdown of ANRIL inhibited osteogenic differentiation by sponging miR-7 through activating the NF-*κ*B signaling pathway, suggesting that ANRIL contributes to periodontitis.

## Data Availability

The data used to support the findings of this study are available from the corresponding author upon request.

## Abbreviations

ALP: Alkaline phosphatase
DMEM: Dulbecco's modified Eagle's medium
lncRNA: Long noncoding RNA
miRNA: MicroRNA
PDL: Periodontal ligament
mut: Mutant
NC: Negative control
PDLCs: Periodontal ligament cells
qPCR: Quantitative polymerase chain reaction
WT: Wild type.

## References

[1] Fischer R. G., Lira Junior R., Retamal-Valdes B. (2020). Periodontal disease and its impact on general health in Latin America. Section V: treatment of periodontitis. *Brazilian Oral Research*.

[2] Genco R. J., Borgnakke W. S. (2020). Diabetes as a potential risk for periodontitis: association studies. *Periodontology 2000*.

[3] Khan S., Barrington G., Bettiol S., Barnett T., Crocombe L. (2018). Is overweight/obesity a risk factor for periodontitis in young adults and adolescents?: a systematic review. *Obesity Reviews*.

[4] Ebersole J. L., Graves C. L., Gonzalez O. A. (2016). Aging, inflammation, immunity and periodontal disease. *Periodontology 2000*.

[5] Cerajewska T. L., Davies M., West N. X. (2015). Periodontitis: a potential risk factor for Alzheimer's disease. *British Dental Journal*.

[6] Fagundes N. C. F., Almeida A. P. C. P. S. C., Vilhena K. F. B., Magno M. B., Maia L. C., Lima R. R. (2019). Periodontitis as a risk factor for stroke: a systematic review and meta-analysis. *Vascular Health and Risk Management*.

[7] Hirashima S., Kanazawa T., Ohta K., Nakamura K. I. (2020). Three-dimensional ultrastructural imaging and quantitative analysis of the periodontal ligament. *Anatomical Science International*.

[8] Zhu Y., Ai R., Ding Z. (2020). lncRNA-01126 inhibits the migration of human periodontal ligament cells through MEK/ERK signaling pathway. *Journal of Periodontal Research*.

[9] Paraskevopoulou M. D., Hatzigeorgiou A. G. (2016). Analyzing miRNA-lncRNA interactions. *Methods in Molecular Biology*.

[10] Huang Y. (2018). The novel regulatory role of lncRNA-miRNA-mRNA axis in cardiovascular diseases. *Journal of Cellular and Molecular Medicine*.

[11] Bhan A., Soleimani M., Mandal S. S. (2017). Long noncoding RNA and cancer: a new paradigm. *Cancer Research*.

[12] Liao K., Xu J., Yang W., You X., Zhong Q., Wang X. (2018). The research progress of lncRNA involved in the regulation of inflammatory diseases. *Molecular Immunology*.

[13] Sánchez-Muñoz F., Martínez-Coronilla G., Leija-Montoya A. G. (2018). Periodontitis may modulate long-non coding RNA expression. *Archives of Oral Biology*.

[14] Ju C., Liu R., Zhang Y. W. (2019). Mesenchymal stem cell-associated lncRNA in osteogenic differentiation. *Biomedicine & Pharmacotherapy*.

[15] Kong Y., Hsieh C. H., Alonso L. C. (2018). ANRIL: a lncRNA at the CDKN2A/B locus with roles in cancer and metabolic disease. *Frontiers in Endocrinology*.

[16] Gholami L., Ghafouri-Fard S., Mirzajani S. (2020). The lncRNA ANRIL is down-regulated in peripheral blood of patients with periodontitis. *Noncoding RNA Res.*.

[17] Li Z., Yu X., Shen J. (2016). ANRIL: a pivotal tumor suppressor long non-coding RNA in human cancers. *Tumour Biology*.

[18] Chen L., Qu H., Guo M. (2020). ANRIL and atherosclerosis. *Journal of Clinical Pharmacy and Therapeutics*.

[19] Chi J. S., Li J. Z., Jia J. J., Zhang T., Liu X. M., Yi L. (2017). Long non-coding RNA ANRIL in gene regulation and its duality in atherosclerosis. *Journal of Huazhong University of Science and Technology. Medical Sciences*.

[20] Thomas A. A., Feng B., Chakrabarti S. (2017). ANRIL: a regulator of VEGF in diabetic retinopathy. *Investigative Ophthalmology & Visual Science*.

[21] Yu L., Qu H., Yu Y., Li W., Zhao Y., Qiu G. (2018). lncRNA-PCAT1 targeting miR-145-5p promotes TLR4-associated osteogenic differentiation of adipose-derived stem cells. *Journal of Cellular and Molecular Medicine*.

[22] Wang Q., Li Y., Zhang Y. (2017). lncRNA MEG3 inhibited osteogenic differentiation of bone marrow mesenchymal stem cells from postmenopausal osteoporosis by targeting miR-133a-3p. *Biomedicine & Pharmacotherapy*.

[23] Xu Y., Qin W., Guo D., Liu J., Zhang M., Jin Z. (2019). lncRNA-TWIST1 promoted osteogenic differentiation both in PPDLSCs and in HPDLSCs by inhibiting TWIST1 expression. *BioMed Research International*.

[24] Zhang N., Hu X., He S., Liu J., Zhang M., Jin Z. (2019). lncRNA MSC-AS1 promotes osteogenic differentiation and alleviates osteoporosis through sponging microRNA-140-5p to upregulate BMP2. *Biochemical and Biophysical Research Communications*.

[25] Piombino P., Riccitiello F., Califano L., Rengo S., Spagnuolo G., Procino A. (2017). The culture medium conditioned by adipose tissue, is able to regulates the lncRNA HOTAIR, HOXC11, HOXC12, Osx and SATB2 gene expression in MSCs during osteoblasts differentiation. *Journal of Stem Cells*.

[26] Spagnuolo G., Procino A. (2018). The lncRNA HOTAIR gene was controlled by polyacrylic acid resins in human gingival fibroblasts and this regulation is time dependent. *International Journal of Clinical Dentistry*.

[27] Liu Y., Zeng X., Miao J. (2019). Upregulation of long noncoding RNA MEG3 inhibits the osteogenic differentiation of periodontal ligament cells. *Journal of Cellular Physiology*.

[28] Zhao J., Sun H., Zhang J. M., Wang M., du X. J., Zhang J. L. (2019). Long non-coding RNA ANRIL down-regulates microRNA-7 to protect human trabecular meshwork cells in an experimental model for glaucoma. *European Review for Medical and Pharmacological Sciences*.

[29] Shu L., Zhang W., Huang C., Huang G., Su G., Xu J. (2020). lncRNA ANRIL protects H9c2 cells against hypoxia-induced injury through targeting the miR-7-5p/SIRT1 axis. *Journal of Cellular Physiology*.

[30] Li G., Gao L., Zhao J., Liu D., Li H., Hu M. (2020). lncRNA ANRIL/miR-7-5p/TCF4 axis contributes to the progression of T cell acute lymphoblastic leukemia. *Cancer Cell International*.

[31] Li M., Pan M., You C., Dou J. (2019). The therapeutic potential of miR-7 in cancers. *Mini Reviews in Medicinal Chemistry*.

[32] Wan S., Wang J., Wang J. (2017). Increased serum miR-7 is a promising biomarker for type 2 diabetes mellitus and its microvascular complications. *Diabetes Research and Clinical Practice*.

[33] Lu P., Wang F., Wu J. (2017). Elevated serum miR-7, miR-9, miR-122, and miR-141 are noninvasive biomarkers of acute pancreatitis. *Disease Markers*.

[34] Akhter R. (2018). Circular RNA and Alzheimer’s disease. *Advances in Experimental Medicine and Biology*.

[35] Zhou X., Jiang L., Fan G. (2019). Role of the ciRS-7/miR-7 axis in the regulation of proliferation, apoptosis and inflammation of chondrocytes induced by IL-1*β*. *International Immunopharmacology*.

[36] Li X., Zheng Y., Zheng Y. (2018). Circular RNA CDR1as regulates osteoblastic differentiation of periodontal ligament stem cells via the miR-7/GDF5/SMAD and p38 MAPK signaling pathway. *Stem Cell Research & Therapy*.

[37] Sun S. C. (2017). The non-canonical NF-*κ*B pathway in immunity and inflammation. *Nature Reviews. Immunology*.

[38] Xue N., Qi L., Zhang G., Zhang Y. (2018). miRNA-125b regulates osteogenic differentiation of periodontal ligament cells through NKIRAS2/NF-*κ*B pathway. *Cellular Physiology and Biochemistry*.

[39] Wang Y. J., Zhang H. Q., Han H. L., Zou Y. Y., Gao Q. L., Yang G. T. (2017). Taxifolin enhances osteogenic differentiation of human bone marrow mesenchymal stem cells partially via NF-*κ*B pathway. *Biochemical and Biophysical Research Communications*.

[40] Zhang B., Wang D., Ji T. F., Shi L., Yu J. L. (2017). Overexpression of lncRNA ANRIL up-regulates VEGF expression and promotes angiogenesis of diabetes mellitus combined with cerebral infarction by activating NF-*κ*B signaling pathway in a rat model. *Oncotarget*.

[41] Xia J., Cao T., Ma C. (2018). miR-7 suppresses tumor progression by directly targeting MAP3K9 in pancreatic cancer. *Molecular Therapy-Nucleic Acids*.

